# Therapeutic Passes and Post-Discharge Outcomes in a Psychiatric Inpatient Unit: A Retrospective Study

**DOI:** 10.1177/07067437221087945

**Published:** 2022-03-15

**Authors:** Natalia Docteur, Emilie Norris-Roozmon, Raphael W. Kusumo, Alex Kiss, Eunice Yixuan Chen, Krista L. Lanctôt, Jay Moss

**Affiliations:** 1 Neuropsychopharmacology Research Group, 282299 Sunnybrook Research Institute, Toronto, ON, Canada; 2 Department of Research Design and Biostatistics, 282299 Sunnybrook Research Institute, Toronto, ON, Canada; 3 Department of Psychiatry, 7938 University of Toronto, Toronto, ON, Canada; 4 Department of Psychiatry, 71545 Sunnybrook Health Sciences Centre, Toronto, ON, Canada

## Introduction

Deinstitutionalization has shifted Canada's mental healthcare system away from prolonged inpatient admission in favor of patient autonomy and community-based treatments. In line with this trend, therapeutic passes are utilized as a risk reduction tool prior to discharge. The rationale and administration guidelines that dictate pass use are often unstandardized, despite their ubiquity in clinical practice.^1^ Proposed justification includes testing treatment efficacy, gauging safety, observing community integration, promoting coping skills, and providing opportunities to socialize.^1–3^ Nonetheless, there is a lack of evidence to support the clinical utility of therapeutic passes in inpatient psychiatry. This study examined the associations between passes and patient outcomes including length of stay (LOS) in hospital, 6- and 12-month inpatient readmissions, and 6-month emergency room (ER) visits post-discharge. The secondary objective was to quantify the longitudinal contribution of passes on ER visits in patients with ≥2 admissions in one year, termed high utilizers (HUs).

## Methods

The retrospective chart review was performed at an academic research hospital affiliated with the University of Toronto. Data were collected from January 1 to December 31, 2017, using a standardized assessment and data collection protocol. Information on passes was extracted from physicians’ notes. Participants included an undifferentiated adult psychiatric sample with a LOS spanning at least 24 h. Pass factors were coded whether they occurred 7 am–4 pm on weekdays, when most clinical services occur, or evenings after 4 pm and weekends, during off-service hours. The Research Ethics Board provided ethics approval and a waiver of consent.

Predictors of psychiatric outcomes were identified to address the primary objective, using Poisson regressions with Pearson chi-square scaling to adjust for overdispersion. For the secondary objective, a linear mixed model (LMM) analyzed significant predictors of ER visits across multiple admissions. Relevant predictor variables selected via literature review were added as covariates. Only significant (*P* < 0.05) variables were retained in the final Poisson and LMM analyses. Analyses were conducted using SPSS v23.

## Results

Four-hundred-ninety-four patients (mean (*M*) age 40.7 years old, 52.7% female) accounted for 596 inpatient admissions. Across total admissions, 405 (68.0%) received ≥1 pass and were administered an average of 3.6 passes with approximately 18.2 hours spent off-unit.

LOS ranged from 1 to 137 days (*M* = 12.2, standard deviation (*SD*) = 15.2). After adjusting for age, psychotic disorders, substance use disorders, and medications at discharge, receiving ≥1 pass, daytime pass hours, and night/weekend pass hours, predicted prolonged LOS, χ^2^(7) = 145.8, *P* < 0.001 (Table 1).

**Table 1.** Poisson Regressions for LOS, Psychiatric Readmissions, and ER Visits Post-Discharge.

	*B*	*SE B*	*Rate ratio*	*P*	*95% CI*	
					*Lower*	*Upper*
*Length of stay* **^a^**						
Intercept	1.243	0.184	3.465	<0.001	2.416	4.969
Age	0.012	0.003	1.012	<0.001	1.006	1.019
Receiving ≥1 pass	0.545	0.136	1.725	<0.001	1.322	2.251
Daytime pass hours	0.020	0.004	1.020	<0.001	1.011	1.029
Night/weekend pass hours	0.004	0.002	1.004	0.020	1.001	1.007
Psychotic disorders	0.301	0.129	1.351	0.020	1.049	1.740
Substance use disorders	−1.139	0.567	0.320	0.045	0.105	0.973
Medications at discharge	0.035	0.014	1.036	0.010	1.009	1.063
*6-month psychiatric readmissions* **^b^**						
Intercept	−1.821	0.137	0.162	<0.001	0.124	0.212
Total number of passes	0.033	0.012	1.033	0.005	1.010	1.057
Medications at discharge	0.049	0.017	1.051	0.003	1.016	1.086
*12-month psychiatric readmissions* **^b^**						
Intercept	−0.874	0.196	0.153	<0.001	0.104	0.226
Night/weekend pass number	0.104	0.032	1.109	0.001	1.043	1.180
Lifetime inpatient admissions	1.062	0.212	2.893	<0.001	1.911	4.381
*6-month ER visits* **^c^**						
Intercept	−1.060	0.109	0.346	<0.001	0.280	0.429
Personality disorders	1.352	0.168	3.866	<0.001	2.780	5.378
Medications at discharge	0.047	0.013	1.048	<0.001	1.022	1.074

^a^ *N* = 344; there were 252 cases missing chart notes for pass hour variables resulting in a reduced sample size for LOS analyses.

^b^ *N* = 585.

^c^ *N* = 592.

Six- and 12-month psychiatric readmissions ranged from 0 to 2 (*M* = 0.25*, SD* = 0.53) and 0 to 4 (*M* = 0.39*, SD* = 0.75), respectively. Total number of passes and medications at discharge were associated with increased 6-month readmissions, *χ*^2^(2) = 19.4, *P* < 0.001. Moreover, number of night/weekend passes and lifetime inpatient admissions predicted higher 12-month readmissions, *χ*^2^(2) = 43.7, *P* < 0.001 (Table 1).

ER visits 6-months post-discharge ranged from 0 to 7 (*M* = 0.54, *SD* = 0.99). No pass factors were associated with this outcome, however personality disorders and medications at discharge predicted increased ER visits, *χ*^2^(2) = 65.7, *P* < 0.001 (Table 1). For the secondary analyses, 70 HUs accounted for 169 unique admissions. HUs had increased ER visits (*M* *±* *SD* = 1.61 ± 2.14) compared to non-HUs (0.42 ± 1.54)*, P* < 0.001. Total number of passes predicted reduced ER visits (*B* *±* *SE* = −0.03 ± 0.01, *t* = −2.63, *P* = 0.021), while medications at discharge were associated with increased visits (0.05 ± 0.02, *t* = 2.77, *P* = 0.004) across multiple admissions.

## Discussion

Overall, passes were associated with poorer post-discharge outcomes including prolonged LOS and increased psychiatric readmissions. Receiving ≥1 pass, daytime pass hours, and night/weekend pass hours accounted for a small but significant amount of variance in LOS. Associations were adjusted for a proxy measure of psychiatric severity, medications at discharge, suggesting that the relationship between pass factors and LOS existed independently from disease severity. Patients were 3.3% more likely to be readmitted after 6-months with each total pass, and 10.9% more likely to be readmitted after 12-months with each night/weekend pass. Our findings align with the positive association between passes and readmissions from a previous chart review,^4^ yet contradict a recent study that found reduced risk of readmission with pass use.^5^ We hope the current cross-sectional findings provide rationale for future prospective investigation on causal relationships between passes and psychiatric readmissions.

No pass factors were associated with ER visits 6-months post-discharge in the whole sample, whereas the total number of passes was associated with reduced ER visits in the HU subgroup. This is a surprising contrast from the whole sample analysis where the same variable was associated with increased readmissions over 6-months post-discharge. Results are difficult to contextualize as there is little evidence on the relationship between pass use and ER visits. However, findings suggest that the clinical utility of passes may differ between utilization groups. Future research should aim to replicate and expand on our findings regarding HUs and therapeutic passes.
